# ENCORE: Software for Quantitative Ensemble Comparison

**DOI:** 10.1371/journal.pcbi.1004415

**Published:** 2015-10-27

**Authors:** Matteo Tiberti, Elena Papaleo, Tone Bengtsen, Wouter Boomsma, Kresten Lindorff-Larsen

**Affiliations:** 1 Department of Biotechnology and Biosciences, University of Milano-Bicocca, Milan, Italy; 2 Structural Biology and NMR Laboratory, Department of Biology, University of Copenhagen, Copenhagen, Denmark; Max Planck Institute for Biophysical Chemistry, GERMANY

## Abstract

There is increasing evidence that protein dynamics and conformational changes can play an important role in modulating biological function. As a result, experimental and computational methods are being developed, often synergistically, to study the dynamical heterogeneity of a protein or other macromolecules in solution. Thus, methods such as molecular dynamics simulations or ensemble refinement approaches have provided conformational ensembles that can be used to understand protein function and biophysics. These developments have in turn created a need for algorithms and software that can be used to compare structural ensembles in the same way as the root-mean-square-deviation is often used to compare static structures. Although a few such approaches have been proposed, these can be difficult to implement efficiently, hindering a broader applications and further developments. Here, we present an easily accessible software toolkit, called ENCORE, which can be used to compare conformational ensembles generated either from simulations alone or synergistically with experiments. ENCORE implements three previously described methods for ensemble comparison, that each can be used to quantify the similarity between conformational ensembles by estimating the overlap between the probability distributions that underlie them. We demonstrate the kinds of insights that can be obtained by providing examples of three typical use-cases: comparing ensembles generated with different molecular force fields, assessing convergence in molecular simulations, and calculating differences and similarities in structural ensembles refined with various sources of experimental data. We also demonstrate efficient computational scaling for typical analyses, and robustness against both the size and sampling of the ensembles. ENCORE is freely available and extendable, integrates with the established MDAnalysis software package, reads ensemble data in many common formats, and can work with large trajectory files.

This is a *PLOS Computational Biology* Software paper

## Introduction

Proteins are dynamical molecules, and the way a protein moves may have a large impact on its function. In addition to determining the “average” structures of proteins, it is therefore becoming increasingly important to describe their structural heterogeneity and dynamics. That is, instead of considering only a single molecular structure of a protein, one should instead represent the entire distribution, or *ensemble* of structures, as representing the structural state [1–3]. As an immediate consequence, comparing molecular states now becomes a question of comparing structural ensembles, rather than individual conformations.

When comparing two structures of a protein one typically calculates the root-mean-square-deviation (RMSD), or one of the alternative scores that quantify the similarity between individual structures [4]. In contrast there is a scarcity of methods for comparison of structural ensembles. The problem was originally addressed by Brüschweiler, who proposed a generalization of the standard single-conformer RMSD technique [5]. A later study by Lindorff-Larsen and Ferkinghoff-Borg defined the problem in terms of comparing the probability distributions that underlie the ensembles, and proposed three different approaches for calculating ensemble similarities [6]. More recently, variations of these methods have been proposed [7–11]. Alternative approaches compare conformational ensembles by directly quantifying similarities in experimental data that report on their structural features [12,13].

Thus, although there are now a few different methods for comparing structural ensembles, it is unclear which to use under which circumstance. Further, most algorithms are not easily accessible in publicly available and user-friendly software, and are not straightforward to implement efficiently. This paper addresses these issues by describing freely available, easy-to-use, efficient implementations of a range of existing ensemble comparison methods. The library, called ENCORE (http://encore-similarity.github.io/encore), interfaces with the MDAnalysis molecular analysis toolkit [14] and can be used both as a Python library and from the command line. The computational efficiency ensures that ensembles with tens of thousands of structures can be dealt with efficiently.

After presenting the design and implementation of the software, we demonstrate how it may be used to examine three problems in structural and computational biology. First, we analyse a set of recently published long molecular dynamics (MD) simulations of ubiquitin and the third IgG-binding domain of protein G (GB3) that were performed with eight different force fields [15]. ENCORE makes it possible to make detailed statements about similarities and differences between the structural dynamics induced by different force fields. Second, by calculating the similarity of ensembles generated with simulations of different lengths we study the important issue of how an MD simulation progresses towards its final distribution of conformations. Finally, using ubiquitin as a well-studied model system, we demonstrate that ENCORE can be used to compare experimentally determined structural ensembles, including those that explicitly aim to model the structural heterogeneity of proteins. Our results show that several different experimentally derived ensembles of ubiquitin are rather similar, and that MD simulations can in certain cases provide ensembles that are very similar to those obtained from experiments. Together with a demonstration of the computational scalability, the results illustrate how the ENCORE software can be used to study problems in computational structural biology.

## Design and Implementation

Molecular ensembles come in different shapes and sizes. Traditionally, Nuclear Magnetic Resonance (NMR) derived experimental structures are typically represented by 10 or 20 structures that individually aim to satisfy all or most of the experimental restraints, and it has been argued that a similar practice should be used in crystallography [2]. Methods such as dynamic ensemble refinement [1], which aim to capture both the structure and dynamics of a protein, have provided ensembles on the order of 100 structures or more [16]. On the other hand, an MD simulation trajectory can also be considered as an ensemble, and might contain thousands if not millions of structures. Indeed, a recent 1-ms simulation of the protein BPTI was saved at a rate of 1 frame per 250 ps of simulation time and generated 4 million individual protein structures [17]. The formats used for such data also vary. While smaller ensembles are often reported in the PDB format, trajectory data are typically stored in more compact, binary formats, and typically depend on the simulation program used. When designing software in this field it is important to take into account the heterogeneous formats in which ensembles are represented.

Another obstacle is the lack of consensus about the algorithms used for conducting ensemble comparisons. The methods that have been proposed for this problem are based on different assumptions, and their relative abilities to describe relevant conformational similarities will depend on the degree of fluctuations in the ensemble. For molecules undergoing minor structural fluctuations, a harmonic assumption might be justified, in which case a simple algorithm for ensemble comparisons can be applied. For larger scale motions, more elaborate and computationally expensive approaches are needed.

The design of the ENCORE software package was driven by a desire to take into account both of the problems outlined above. First, the software tool is built on MDAnalysis, a well-established software framework for dealing with different molecular file formats, and can thus automatically deal with a variety of inputs. Second, we make available a number of different comparison algorithms within a single framework, facilitating the process of comparing ensembles with different algorithms.

**Fig 1:**
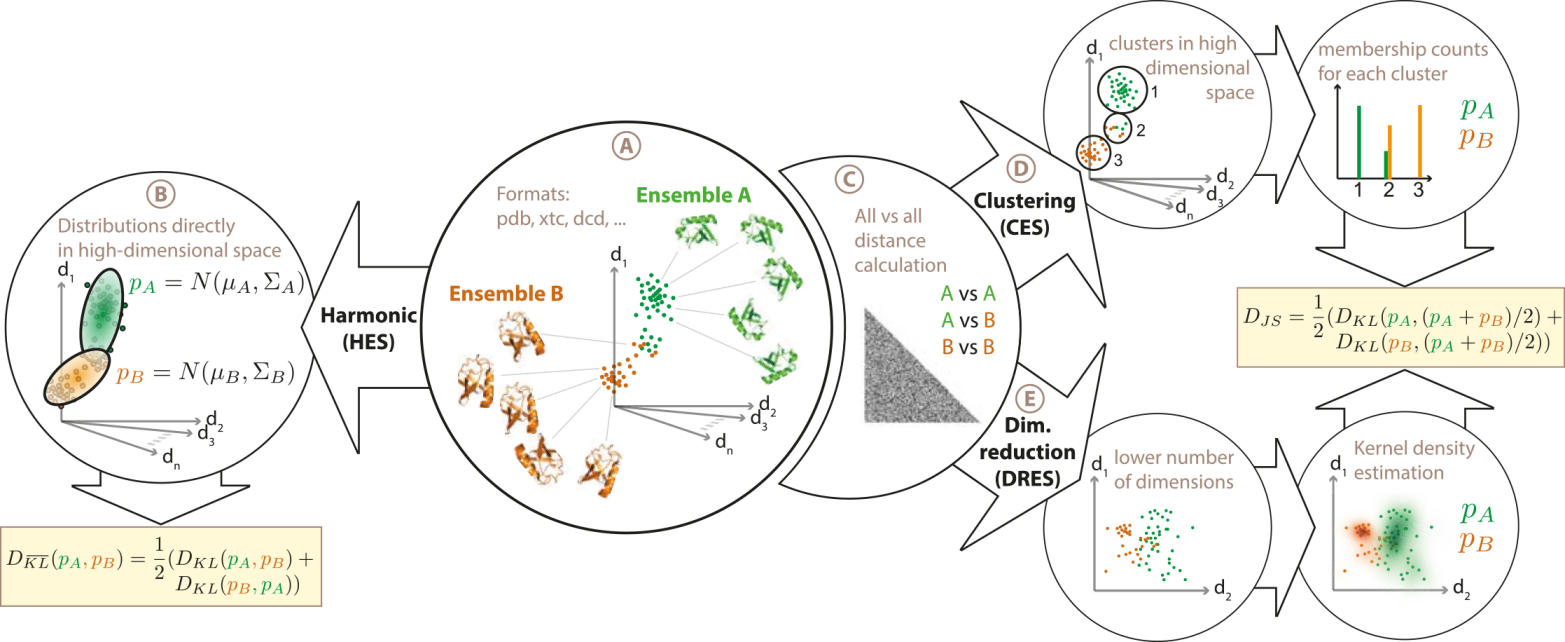
Overview of ensemble comparison methods implemented in ENCORE. ENCORE implements three different methods for ensemble comparison. (A) As input ENCORE takes two or more conformational ensembles in a number of formats including PDB files and various trajectory formats. (B) In the harmonic ensemble similarity method (HES) each ensemble is represented as a high-dimensional Gaussian distribution, N(μ,∑) whose mean (μ) and covariance matrix (∑) ENCORE estimates from the conformational ensembles. A similarity score is then calculated as a symmetrized Kullback-Leibler divergence ($D_{\bar{KL}}$) between each pair of probability distributions. (C) In the calculation of the two other similarity measures, the first step is to calculate the pairwise RMSD between all structures in all ensembles. In ENCORE, this step can be parallelized over multiple computing cores and the results can be stored on disk for additional later analyses. (D) In the clustering-based ensemble similarity (CES) method, the matrix of pairwise RMSDs is used as input to the Affinity Propagation algorithm to cluster the structures from all ensembles together. The cluster populations from each ensemble are then used as basis for calculating the Jensen-Shannon divergence (*D*_JS_) as a measure of ensemble similarity. (E) In the dimensionality-reduction-based ensemble similarity (DRES) method, the matrix of pairwise RMSDs is used as input to the Stochastic Proximity Embedding algorithm to project the high-dimensional conformational ensemble into a low-dimensional space. Using a kernel density estimation method in this lower dimensional space, ENCORE creates probability distributions for each ensemble, which are used as basis for calculating the Jensen-Shannon divergence between the ensembles. See ref. [6] for additional details on the three ensemble similarity methods.

Currently, three comparison algorithms are available within ENCORE. These have been described in detail elsewhere [6], and will only briefly be discussed here (Fig 1). Underlying all three methods is the idea that we first use the structural ensemble to estimate the underlying probability distribution, and then quantify the similarity of two derived probability distributions using symmetrized versions of the Kullback-Leibler divergence measure. The three methods, which mostly differ in how the probability distributions are derived, are: 1) A fast harmonic algorithm for small-scale fluctuations (HES: harmonic ensemble similarity), 2) A structural clustering based method (called CES), where similarity is defined by overlapping membership in the clusters, and 3) a dimensionality reduction method (called DRES), where similarity is defined through projecting the ensembles into lower dimensional spaces. The basic algorithms have been presented before [6], and we here focus on a completely new, user-friendly and efficient implementation aimed to make the methods widely available and extendable.

The ENCORE software has a modular structure, so that the main steps in the calculations are independent, making it easy to extend and improve the software, as well as to use the modules independently. Most of the control flow and the general structure are written in Python for convenience, while the time-consuming parts are written as C libraries which are called by Python through Cython wrappers [18]. For example, the software contains C-implementations of the Affinity Propagation clustering [19] and Stochastic Proximity Embedding (SPE) projection [20] algorithms. The software is designed to make optimal use of fast multi-core machines in order to speed-up time-limiting operations. The currently implemented clustering-based and the dimensionality-reduction-based methods rely on the availability of a matrix of conformational similarity or distance values (e.g. RMSD), and obtaining this distance matrix can be the main bottleneck in the calculations. Our code therefore executes this calculation on multiple computational cores (with essentially linear-scaling), and the matrix can also be saved conveniently to disk to facilitate multiple analyses on the same system.

## Examples of Use of ENCORE

To provide examples of how ENCORE might be used in structural biology research and to demonstrate the general applicability of the software, we provide examples of three different use-cases. The first demonstrates how ensemble comparisons can be utilized to quantify the differences between simulations with different molecular force fields. The second is concerned with assessing convergence in molecular simulations. Finally, we conclude with a comparison of different experimentally derived structural ensembles for the same molecular system. For these examples we have selected previously examined protein systems to ease comparison with earlier observations.

### Comparing force fields

**Fig 2:**
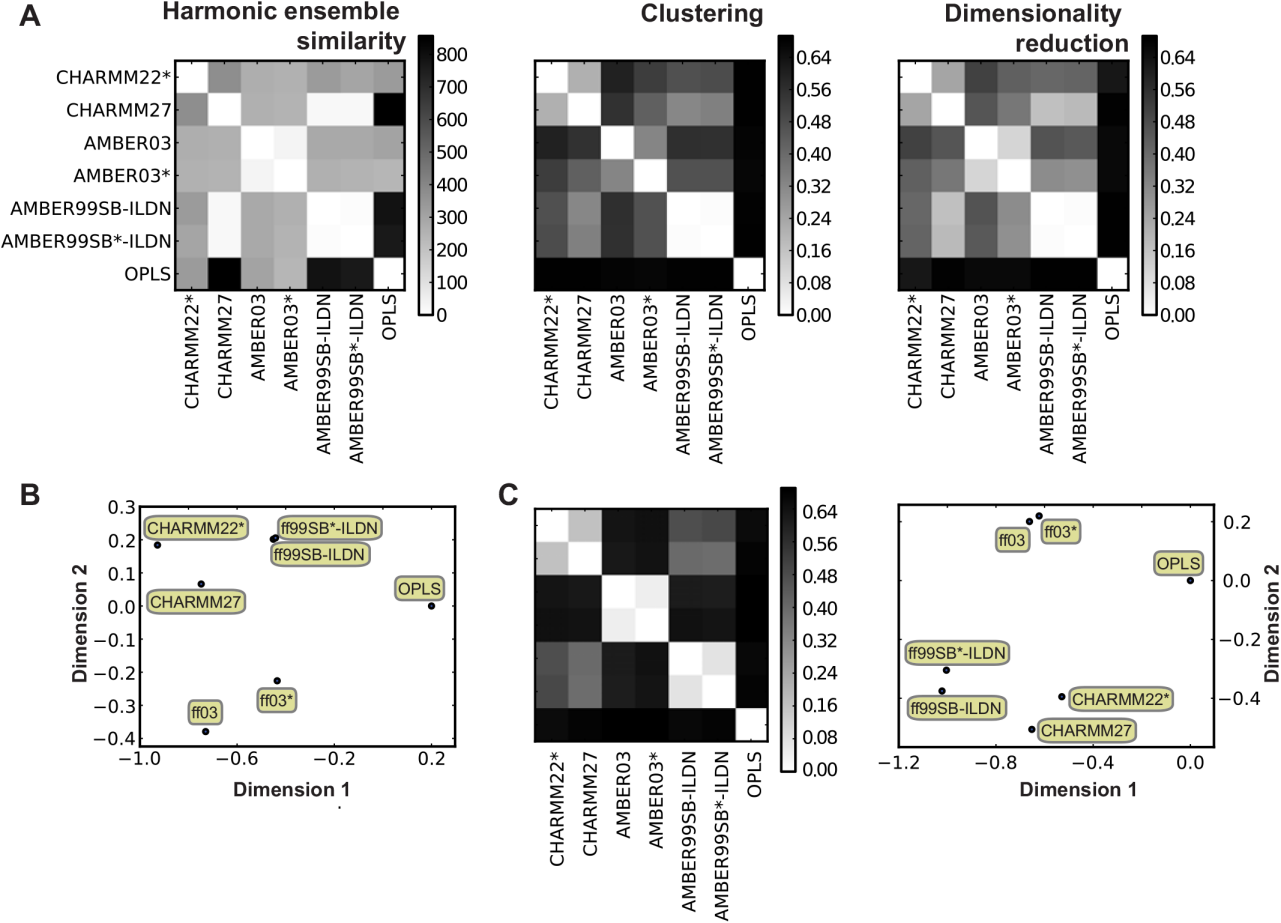
Comparing molecular simulations using ENCORE. We used ENCORE to calculate the similarity between seven molecular dynamics simulations of (A, B) protein G and (C) ubiquitin. (A) The plots show the pairwise similarity between the seven ensembles computed using the three different ensemble comparison methods. (B) Using the similarities calculated by the CES method as an example, a tree-preserving embedding method was used to represent the ensembles in two-dimensions. In this plot, the distance between pairs of ensembles mimics (to the extent possible in two dimensions) the similarity between different ensembles. In agreement with the pairwise similarities, three pairs of ensembles (CHARMM22*/CHARMM27, ff99SB-ILDN/ff99SB*-ILDN, and ff03/ff03*) are located relatively close to one another, in line with the similar origins of each pair of force fields. (C) We performed similar calculations on seven ubiquitin simulations, again using the CES method as an example and projecting the similarities into two-dimensions. A similar organization is found for the different ensembles for both proteins, as is also evident from directly comparing the matrices of ensemble similarities. Note that in the projections, the axes have no direct physical meaning beyond their scale, which are determined so that the distance in the projections are close to the calculated *D*_JS_. Note also that since these distances are invariant to rotations, translations and inversions of the projections, it is the relative positions in the two plots that should be compared.

When performing molecular simulations it can be difficult to interpret how the choice of a force field affects the resulting structural ensembles. Although the differences are well described at the parameter level, the structural consequences for a particular system are hard to predict. Sometimes it may be necessary to conduct simulations with different force fields, but even when such simulations have been completed, it is not trivial to quantify the observed differences. The software presented in this paper facilitates this process. As an illustration, we analyse a set of recently published 10-μs MD simulations on ubiquitin and GB3, using eight force fields from the CHARMM, AMBER and OPLS families [15]. We calculated the ensemble distances between each pair of trajectories, using 1000 frames from each trajectory (Fig 2A presents the results for GB3). Note that for both proteins, the CHARMM22 trajectories were excluded from these figures, as the structure substantially deteriorated during those simulations and their inclusion would therefore mask the remaining comparisons.

As an aid to further interpret and visualize the pairwise similarities between the seven ensembles, we projected the results into two dimensions using a tree preserving embedding method [21] (Fig 2B). In this plot, the distance between two ensembles represents (as well as possible in a two-dimensional projection) the similarity between the corresponding ensembles (here represented by the clustering-based method). Such an analysis provides a convenient overview of the relative similarity of the different ensembles, in the same way as e.g. multidimensional scaling [22] or non-linear projection methods [23] can be used for comparing multiple static structures. Given the different approximations involved in the three methods for ensemble comparison that are currently implemented in ENCORE it is instructive to compare the resulting similarities. We find a good quantitative agreement between the clustering (CES) and dimensionality reduction (DRES) methods, while the harmonic method (HES) is in qualitative agreement (Fig 2). Common to the three methods is that certain pairs of trajectories, in particular Amber ff9SB-ILDN/ff9SB*-ILDN and ff03/ff03*, but also CHARMM22*/CHARMM27, are closer to one another than to the remaining simulations. Also, all methods show clearly that the simulation performed with OPLS differs the most from the remaining six ensembles. Very similar results are obtained when analysing an equivalent set of ubiquitin simulations (Fig 2C).

The results also highlight the fact that the HES scale is different from the DRES and CES scales. This is due to the fact that HES uses a different symmetrized version of the Kullback-Leibler divergence than the other two methods. Thus, while the Jensen-Shannon divergence used in DRES and CES may take on values between zero and ln(2)~0.69, HES can in principle take on values between zero and infinity. We note also that while our choice of Kullback-Leibler-based scores is inspired by its origin in information theory, an alternative interpretation of the divergence measure is as a (non-equilibrium) free-energy difference between the two ensembles [24,25]. The basis of the similarity scores in both thermodynamics and probability theory also highlights that any differences between ensembles that are not manifested in (equilibrium) distributions of the conformations (e.g. changes in the dynamics or rates of transitions between states) will not be captured.

The similarity of ensembles generated with particular pairs of force fields in Fig 2 (e.g. ff99SB-ILDN and ff99SB*-ILDN) is interesting and highlights an important observation on the relationship between force field parameterization and the generated ensembles. These pairs of force fields, whose names differ by the inclusion of an asterisk, differ mostly in the parameters that define the backbone torsion angle potentials. In particular, the force fields ff99SB*, ff03* and CHARMM22* were derived from ff99SB, ff03, and CHARMM27 by modifying the backbone potential to give rise to a more accurate description on the energetic balance between extended and helical structures [15]. Our finding that the structural ensembles obtained from the 10 μs long MD simulations are very similar despite these important modifications suggests that simulations of this length are not sufficiently long and have too few transitions between distinct backbone conformations to expose the force field differences, which we expect would otherwise have given rise to more dissimilar ensembles. This observation in turn suggests that simulations with the level of sampling presented here are not alone sufficient to parameterize and test new force fields.

We recently analysed these same trajectories with a standard principal component (PC) analysis [26]. The results presented here are fully consistent with those results providing an overall validation of the observations from ENCORE. The current results, however, provide some additional insights. For instance, projections on the first PCs showed that simulations performed with ff99SB-ILDN, ff99SB*-ILDN, CHARMM22* and CHARMM27 cover relatively similar and well-defined structural ensembles, while simulations with ff03 and ff03* sample distinct, but also relatively well-defined regions of the conformational space. Based on the PC analysis and the calculation of the root mean square inner product using the first 10 PCs it was, however, more difficult to discern any differences between the ensembles generated with ff99SB-ILDN, ff99SB*-ILDN, CHARMM22* and CHARMM27. In contrast, the results obtained by ENCORE show clearly that, for both GB3 and ubiquitin, each pair of force fields (ff99SB-ILDN/ ff99SB*-ILDN, ff03/ff03*, CHARMM27/CHARMM22*) gives rise to ensembles that are more similar to one another than to ensembles from any of the remaining force fields. Further, we note that while quantities such as the root mean square inner product [27] can provide very useful information about the similarity between the conformational space spanned by ensembles, such methods are less directly affected by variations in the distributions of conformations within these spaces. The PC analysis also revealed that some ensembles appear to sample multiple similar, but distinct structural states. This means that one can only approximately describe these ensembles as normally distributed, which can explain some of the differences between the harmonic ensemble similarity method and the results from clustering and projections (Fig 2).

ENCORE provides the option for assessing the uncertainty of the calculated similarity scores using a bootstrap procedure, which conducts repetitions of the calculations on sub-sampled ensembles. In particular, for each ensemble we sampled (with replacement) a new ensemble with the same number of conformations and calculated the divergences between these resampled ensembles. This procedure was repeated many times and the uncertainties of the divergence measures were calculated as the variance over these estimates. As an example of the method and the level of uncertainty found, we calculated uncertainties for representative entries in Fig 2 (Table 1). In all cases we found the uncertainties to be rather small (average relative error is 6% with a maximum of 19%), with in particular the HES and CES methods appearing particularly robust. In all cases we find that the differences observed between the different ensembles are substantially larger than the noise level inherent to the procedure. We also analysed ten times smaller ensembles and found the relative error to increase on average by a factor of 3.6 (see also further below on an analysis of the robustness of the divergence measures towards sparsification).

**Table 1:** Calculating uncertainties of similarity scores using a bootstrap procedure. The uncertainties were calculated for representative entries from Fig 2 as standard deviations over 100 bootstrapped subsamples of the ensembles.

	d_HES_	d_CES_	d_DRES_
Amber03 vs. Amber03*	66.0±0.1	0.33±0.01	0.18±0.03
Amber03 vs. CHARMM22*	388.7±0.4	0.61±0.01	0.39±0.05
Amber03* vs. CHARMM22*	364.5±0.3	0.53±0.02	0.23±0.04

### Assessing convergence

Assessing convergence is crucial when performing molecular simulations or protein structure determination, since only when fully converged does the ensemble actually represent the probability distribution (e.g. Boltzmann distribution of a force field) that one aims to sample. While an exhaustive assessment of the convergence of a simulation is difficult, because there might be events occurring on longer time scales than the one probed in simulation [28], it can be valuable to evaluate the degree of convergence within the visited conformational basins [29]. The ensemble comparison methods in ENCORE can be used for this task e.g. by quantifying the difference between the full trajectory and time-windows of increasing size. In this way we can answer the question of how quickly a simulation converges to our current best estimate of the complete distribution of conformations (i.e. the entire simulation). This idea is conceptually similar to the commonly used root-mean-square-inner-product [30], but differs in that we not only measure the overlap of the conformational spaces that are sampled, but also more directly quantify the extent to which the populations agree.

**Fig 3:**
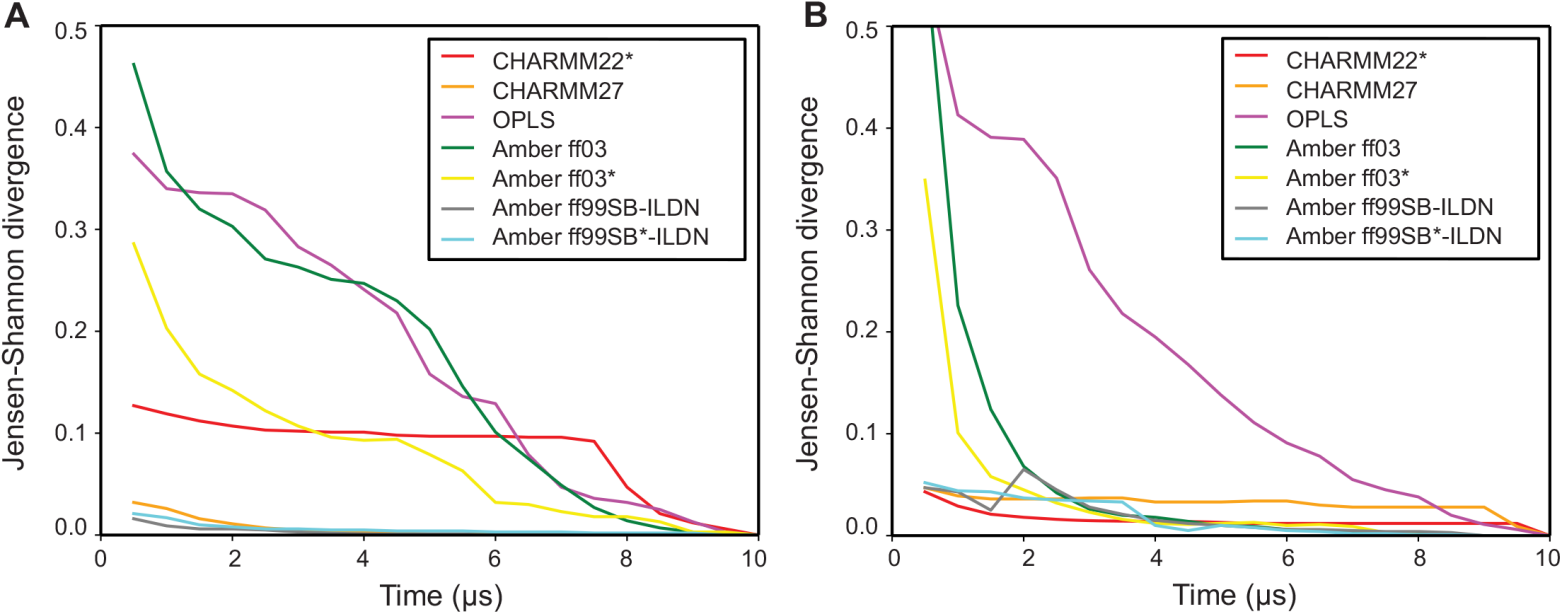
Assessing the rate of convergence in molecular simulations. Using the CES score we used ENCORE to assess the rate of convergence in seven molecular dynamics simulations of (A) protein G and (B) ubiquitin. In each case, we compared simulations of increasing length to the full ensemble obtained after 10μs of simulation. Per definition, the similarities thus decrease to zero at 10μs, but the rate at which low-values are obtained indicates how quickly the simulations have reached a distribution of conformations that is similar to the full ensemble. For example, simulations of both proteins with Amber ff99SB-ILDN and ff99SB*-ILDN quickly drop to very low values, reflecting the fact that the ensembles obtained after a few microseconds are very similar to those obtained at the end of the simulation. In contrast, for example, simulations with OPLS continue to explore new regions of conformational space during the entire simulations.

We illustrate this approach on the trajectories from the previous section, by calculating the ensemble similarities between the full simulations and shorter segments of increasing sizes (Fig 3). By construction, all curves eventually converge to zero, but the shape of the curve provides information about how quickly the simulation starts revisiting states it has already seen. For instance, for both GB3 and ubiquitin the trajectories of CHARMM27, AMBER ff99SB-ILDN and AMBER ff99SB*-ILDN very quickly drop to a low ensemble divergence, showing that these simulations quickly cover the same region of conformational space as the entire 10-μs simulation. The simulations performed with AMBER ff03* (and to some extent also the ff03 simulations) take slightly longer to reach a similarly low level of divergence to the full simulation. This can be understood in terms of the discussion above and our previous PC analysis [26], which show that these simulations sample a relatively well-defined state that is slightly different from that sampled in the more accurate (as evaluated by comparison to experimental data [15]) simulations with e.g. ff99SB*-ILDN. Thus, since the simulations are initiated from an experimental structure, they first relax into this slightly shifted conformational basin, and then have to explore that state to reach convergence. Finally, for both proteins the convergence analysis of the simulations performed with OPLS shows ensemble similarities that decrease monotonically without reaching a plateau, showing that they continue to explore new conformations. These observations are fully consistent when using both the CES and DRES methods.

Although the convergence assessment procedure as described here can compare only single trajectories, there are various ways in which it can be extended to support multiple-trajectory simulations (such as those arising in distributed computing or replica-exchange simulations). One simple approach is to concatenate the individual trajectories into a target ensemble, and then measure its similarity to sub-ensembles of increasing size. For replica exchange or replica-averaged simulations, where different replicas are often constructed such as to sample the same distribution after convergence, it can also be useful to compare the ensembles obtained by the different replicas as the simulation progresses, to verify that all replicas have sampled the same conformational space.

### Comparison of experimentally-derived ensembles

In addition to ensembles generated e.g. by simulations using transferable molecular force fields, it is also becoming increasingly common to generate structural ensembles through a more direct integration of experiments and simulations. Most protein structures that are deposited in the Protein Data Bank have been generated to provide a single conformation that best fits all the available experimental data. (We note that this is also the case for most NMR ensembles, typically consisting of e.g. 10–20 structures, where each structure represents different but equivalent solutions to the optimization problem). Using combinations of experiments and simulations in methods such as ensemble refinement [31], time-averaged restrained simulations [32], Bayesian [33,34] or maximum entropy [35–38] methods one can, however, determine conformational ensembles to represent the structural dynamics of a protein. In short, these methods aim to derive structural ensembles that are simultaneously compatible with the experimental data at the ensemble level and prior structural information, typically encoded in a molecular force field. With increasing quality of the experimental data, molecular force fields [15] and theoretical background for such methods [38] it is becoming more common to use these methods, but so far no systematic study has compared the ensembles that result from using different kinds of experimental data and refinement approaches. We here demonstrate how ENCORE has allowed us to compare structural ensembles obtained by computation, experiments or through the combination of the two.

In the case of human ubiquitin, several ensembles have been generated using different kinds of experimental data. In addition to providing an atomic level view of the structural dynamics of a protein, these studies have provided insight into the role protein motions may play in function. In particular, a collection of several ubiquitin crystal structures, a so-called HSP ensemble, displays conformational variability similar to that revealed by NMR experiments on free ubiquitin in solution [39]. Similarly, a structural ensemble of free ubiquitin derived using NMR data displays a substantial overlap with an HSP ensemble [40]. Together, these studies suggest that ubiquitin free in solution samples an ensemble of conformations that may be “predisposed” towards the different structures this protein can take when binding to its various biological targets.

**Fig 4:**
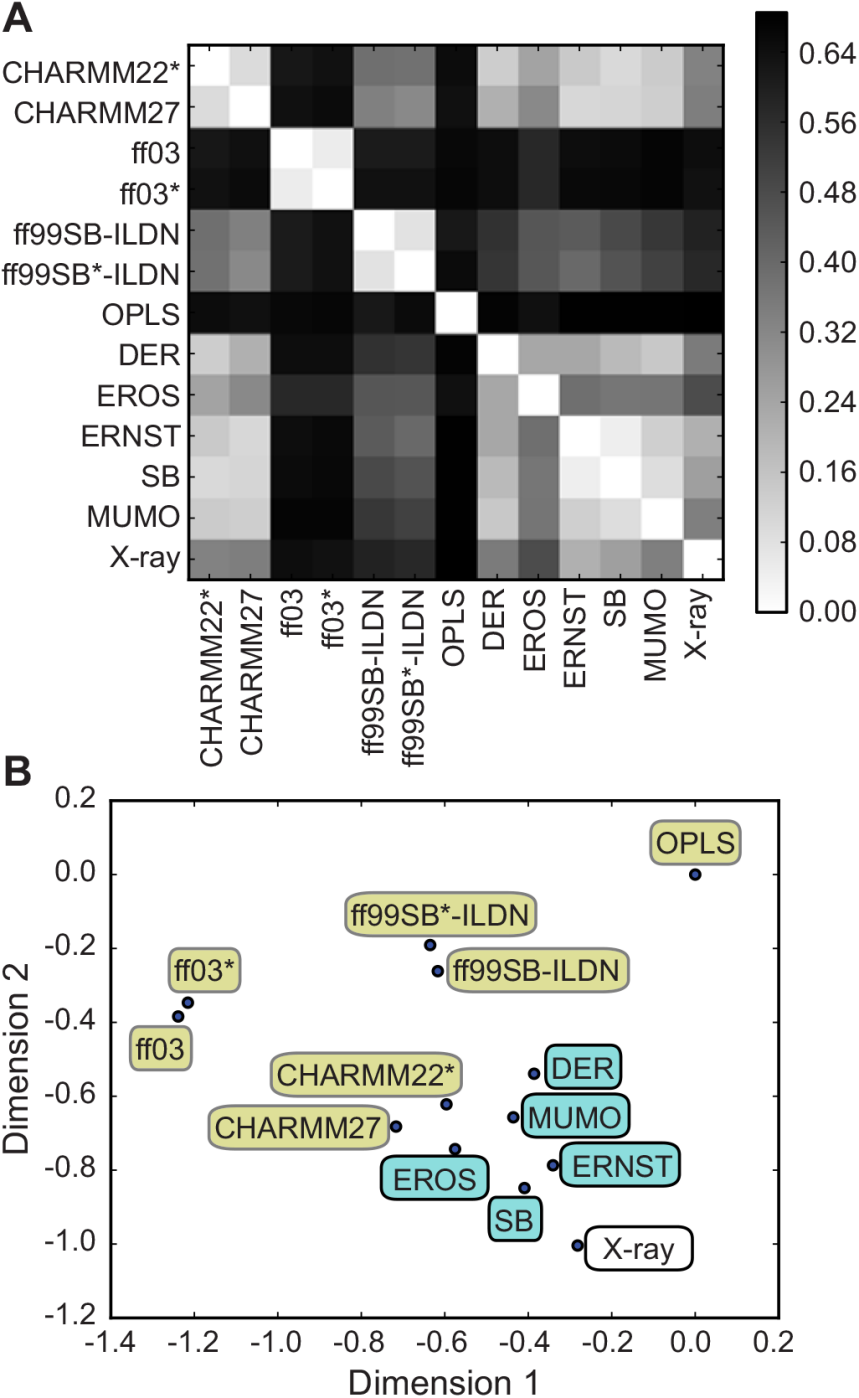
Comparing ubiquitin ensembles from simulations and experiments. We used ENCORE to compare 13 previously determined conformational ensembles of human ubiquitin: seven ensembles were obtained by molecular dynamics simulations with different force fields, five ensembles were generated via replica-averaged simulations that used experimental NMR data as restraints in molecular simulations and a single ensemble was obtained as a collection of 46 different crystal structures of ubiquitin. (A) Using CES we calculated the pairwise similarity of all 13 ensembles and (B) projected the results into two dimensions. Note how the molecular simulations (yellow labels) result in a broader range of conformational ensembles whereas the ensembles restrained via different kinds of experimental NMR data (blue labels) are all more similar to one another. This observation is evidence of the fact that experimental restraints, when used in replica-averaged simulations, can be thought of as system specific correction to the energy function used, which guides the simulations towards the correct conformational ensemble. Finally, note how the NMR-restrained simulations are also relatively similar to a collection of ubiquitin X-ray structures. This observation reiterates the notion that ubiquitin in solution samples a conformational ensemble that is similar to the variability observed in different ubiquitin structures, and also that such ensembles can be derived relatively robustly by combining NMR data and molecular simulations. Importantly, the five NMR ensembles were obtained using different procedures, force fields and sources and types of experimental data.

Using ubiquitin as an important model system for studying the structural dynamics of proteins by integrated computational and experimental methods, we here used ENCORE to compare the various structural ensembles of this protein. In particular, we compare thirteen different ubiquitin ensembles: (i) five ensembles determined using different methods for ensemble refinement using NMR data [1,16,40–42] (ii) seven ensembles obtained using molecular dynamics simulations [15] and (iii) an HSP ensemble consisting of 46 different crystal structures of ubiquitin [40]. Using the CES method implemented in ENCORE we calculated the pairwise similarity between all 13 ensembles (Fig 4A) and projected the results in to two dimensions for easier visualization (Fig 4B).

The results provide interesting new insight into the conformational ensembles obtained by simulations and experiments. Focusing first on the seven ensembles obtained from MD simulations we find the same overall pattern as obtained above (Fig 2) when these ensembles were analysed alone. Thus, we find that the similarities calculated by ENCORE in this case are not sensitive to the inclusion of the additional ensembles determined by experiments. Looking at the five ensembles obtained by ensemble refinement using e.g. NOEs and relaxation order parameters (DER, MUMO) or using multiple sets of residual dipolar couplings (EROS, ERNST, SB) we find that these ensembles cluster together in a smaller region of conformational space. Thus, it appears that the experimental data indeed can act as system-specific corrections to the energy function used for sampling that give rise to a relatively well-defined solution independent of the both protocol and data. In particular, we note that in addition to the differences in the kinds of data that were used and the way they were integrated with simulations, the different ensembles involved different molecular force fields (DER, MUMO: CHARMM22, SB: Amber ff99SB and EROS: OPLS). Thus, the results that we obtained using ENCORE lead to the conclusion that ensembles obtained by refining conformations against experimental NMR data, when compared to MD simulations, more robustly represent the structural dynamics of ubiquitin.

Of considerable interest we also find that these experimentally refined ensembles in general are close to the X-ray HSP ensemble. This observation strengthens further the conclusion that ubiquitin free in solution samples an ensemble similar to that sampled by ubiquitin when bound to different binding partners [39,40]. Our observation that the experimental data act to restrain the motions observed in the unbiased MD simulations to a more well-defined ensemble, similar to that found in crystal structures, is in line with recent findings based on an analysis of independently measured residual dipolar couplings [43]. Finally, we note that the MD ensembles obtained using either CHARMM27 or CHARMM22* fall within the same region of conformational space as the experimentally determined ensembles (none of which used these two force fields in their refinement) and are also relatively close to the HSP ensemble. These two ensembles were also previously found to provide the best agreement with experimentally measured residual dipolar couplings in ubiquitin [15].

## Performance

Given the tendency towards larger ensembles and trajectories being reported in the literature, ENCORE was designed with efficiency in mind, with the explicit goal of making ensemble comparisons with tens of thousands of structures possible as a routine task. The computational simplicity of the HES method makes it naturally scalable to large ensembles. However, both the CES and DRES ensemble methods rely on the calculation of an RMSD distance matrix, which was found to be prohibitively costly for large ensembles. To improve scaling for these ensemble-comparison methods the RMSD calculation was implemented to take advantage of multiple processing cores in parallel. In addition, all clustering and dimensionality reduction functionality was implemented in C.

**Fig 5:**
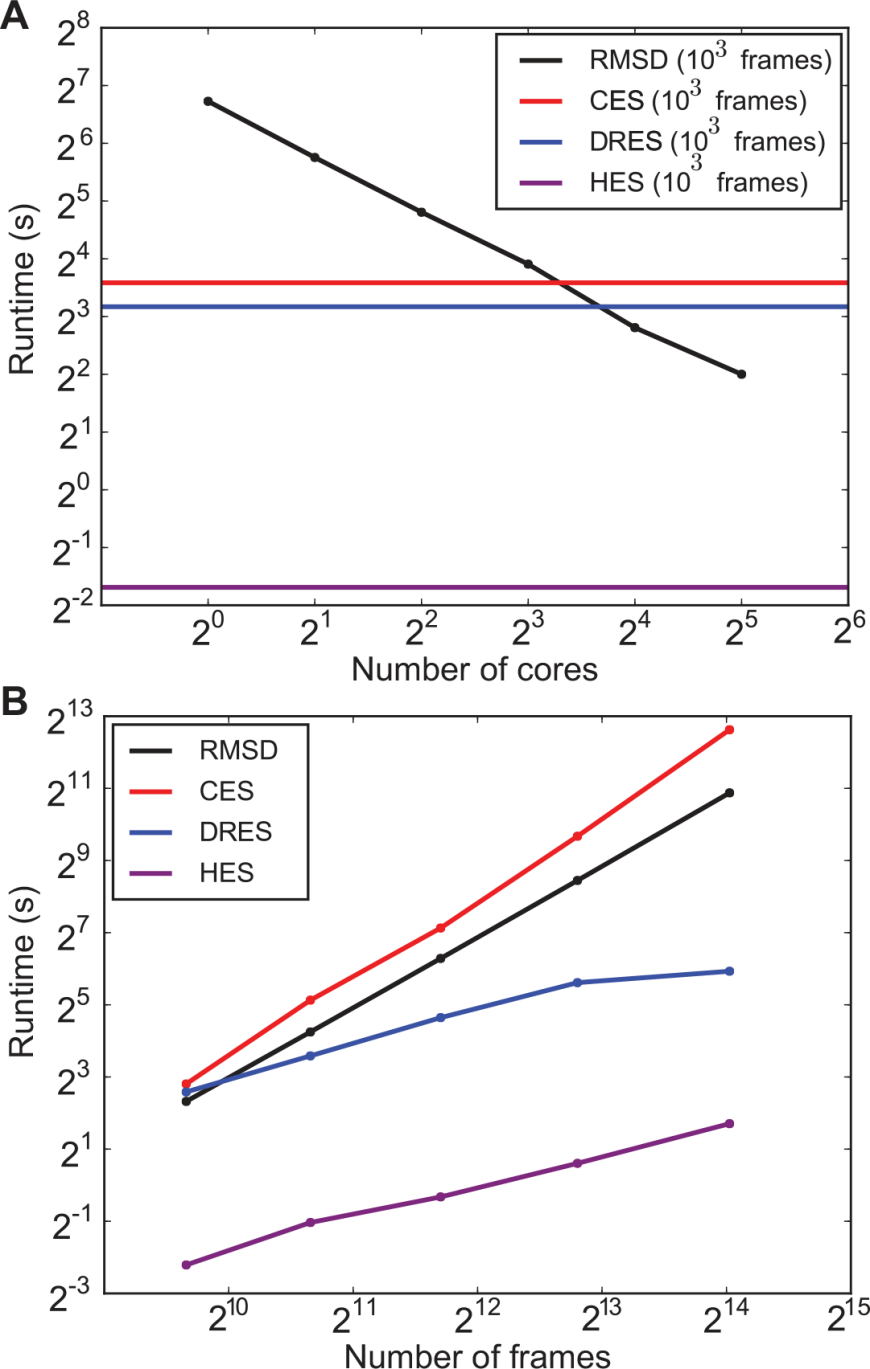
Computational scaling of ENCORE calculations. We determined how the runtime of ENCORE scales with (A) the number of computer cores when executing ENCORE in a parallel fashion and (B) when varying the number of frames for a fixed number of computer cores. In (A) the black line illustrates how the pairwise RMSD calculation can be sped up by distributing the calculations over multiple cores. As the clustering and dimensionality reduction methods that have so far been implemented have not been parallelized they appear as horizontal lines that will eventually (above 8–16 cores) limit the overall calculations. In (B) we show how the overall runtime increases for both the RMSD calculations, clustering and dimensionality reduction as the number of frames are also increased. We used 16 processor cores for the calculations.

We probed the computational scaling behaviour of these critical components in two different ways: 1) the total runtime as a function of the number of processor cores (Fig 5A), and 2) the total runtime as a function of the number of structures in the ensembles (Fig 5B). The former clearly illustrates that the dominating RMSD distance calculation now scales linearly due to parallelized execution on multiple cores. The clustering and dimensionality reduction algorithms have not been parallelized in this first release of ENCORE, but their contribution to the total computational costs can be seen to remain less significant when executing with up to 8–16 cores, which are common values for modern computer architectures. For a fixed number of processing units (16), we see that the clustering itself now dominates slightly with a quadratic complexity, while dimensionality reduction scales a bit better (following the original description, the stress parameter was chosen to scale linearly with ensemble size [20]). In all cases, we find that the simpler HES method is computationally much more efficient both in terms of its run time and the scaling with the ensemble size.

**Fig 6:**
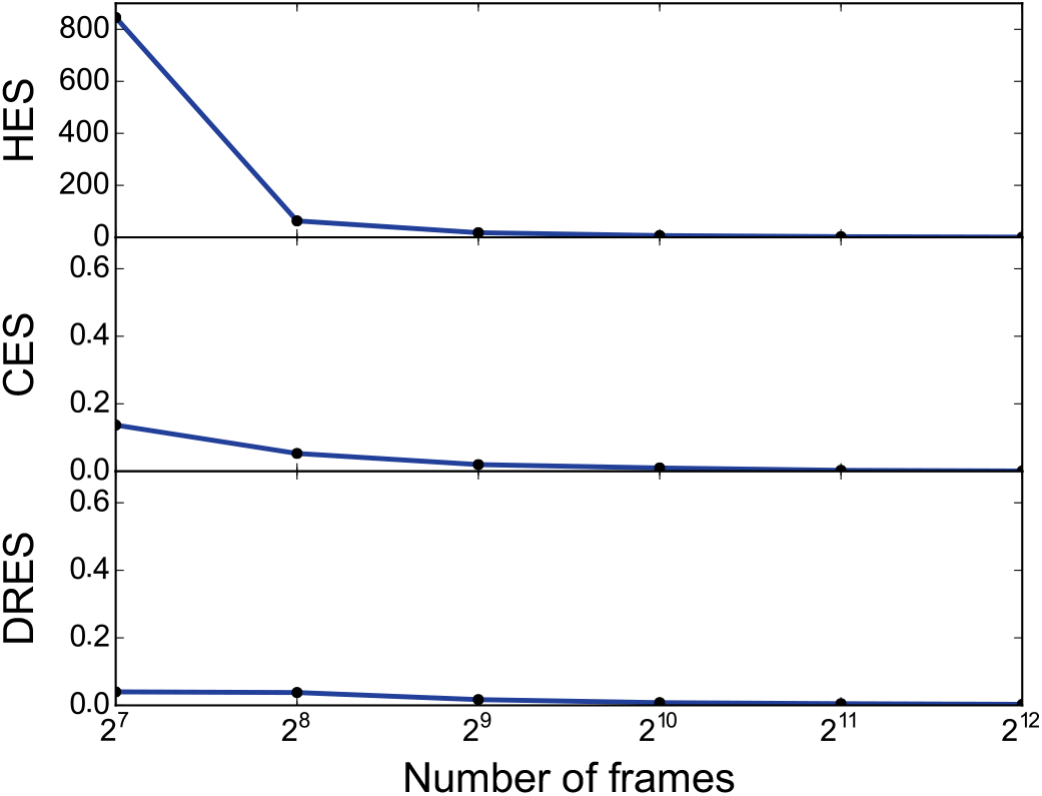
Effect of sparsifying the simulation data. We evaluated the robustness of the calculated similarity scores when decreasing the ensemble size. In particular, we took 8192 (2^13^) frames separated by 1ns from a simulation of GB3 using Amber ff03 as a reference and created subensembles of various sizes by iteratively removing every second frame. We subsequently calculated the three different similarity scores between the full ensemble and the various subensembles that contained between 128 and 4096 frames. The results show that even when only every 16th frame is retained the pairwise similarity is very high (divergence close to zero), demonstrating both the robustness of the calculations and that such sparsification likely is an efficient way of improving computational efficiency in practice.

For ensembles arising as trajectories from simulation, a natural way of increasing the performance of the analysis is to decrease the time resolution by only using every n’th frame of the original ensemble. We investigated the sensitivity of the three similarity methods to such sparsifications of the data by calculating the similarity between the original ensemble and ensembles sparsified to include only a fixed number of (uniformly spaced) frames (Fig 6). The results demonstrate that, at least on the types of ensembles considered here, little is gained by using more than 1000 frames.

## Availability and Future Directions

ENCORE is freely available (http://encore-similarity.github.io/encore), together with its documentation and several examples on how to use it, and is distributed under the GNU general public license, version 3. The code can readily be extended to include additional methods for ensemble comparison as well as for novel uses for the algorithms that have already been implemented. One of the primary directions for future work is to extend the capabilities of the CES and DRES methods to beyond that of tens of thousands of structures. This would require finding alternative methods that do not need the full RMSD distance matrix, since it will neither be feasible to calculate nor store such large matrices in memory. The memory issue can be resolved by supporting on-the-fly distance calculations, or by using landmark-based methods that have previously successfully been used e.g. in projection of conformational ensembles [44]. The complexity of the RMSD calculations itself could be overcome by using alternative distance metrics such as Gaussian Integral based techniques [45]. Finally, for the CES approach, better scaling would require using a sub-quadratic time clustering method. Other future work on ensemble comparison methods could include using mixture models [46] to represent the probability distributions instead of the kernel density estimates currently used in the projection-based method. Also, currently both the clustering and projection based methods use the matrix of pairwise RMSDs to define the overall conformational space. For ensembles generated from simulations of protein folding or for intrinsically disordered proteins it may, however, be more appropriate to use other similarity scores than the RMSD [23,47] and such scores may also readily be implemented in ENCORE. In the case of molecular dynamics simulations, the time evolution of the system provides additional information that can be used e.g. to build Markov state models [48] or in kinetic clustering schemes [17]. It has recently been demonstrated how the Jensen-Shannon divergence provides a natural means to compare two different Markov state models [49] and it will be interesting to determine to what extent the temporal information, when present, helps in comparing molecular ensembles. Also, we note that as it is difficult to provide a simple geometric interpretation of the scores, we suggest they are currently best interpreted in a relative fashion (e.g. ensemble A is more similar to B than to C), and hope that a more widespread application of the methods will help provide a clearer intuitive understanding for the absolute values of the similarity scores. Finally, we hope that the broad availability and easy use of the ensemble comparison methods that ENCORE confers will spur new studies that both use the methods to provide biological insight as well as allow for more systematic comparisons and evaluations of the strengths and weaknesses of the algorithms involved.

## Supporting Information

S1 Software ENCORE software and examples. (GZ)
