# Supplementary material for: ENCORE: Software for Quantitative Ensemble Comparison
Source: PLoS Comput Biol. 2015 Oct 27;11(10):e1004415. doi: 10.1371/journal.pcbi.1004415 (PMC4624683; doi:10.1371/journal.pcbi.1004415)
Supplement: S1 Software — (GZ) [file pcbi.1004415.s001.gz › encore-master/docs/_build/latex/encore.pdf]

ENCORE Documentation

Release 1.0

June 01, 2015

CONTENTS

1 Introduction 1

2 encore package 3
2.1 Submodules . . . 3
2.2 encore.Ensemble module . . . 3
2.3 encore.confdistmatrix module . . . 4
2.4 encore.covariance module . . . 5
2.5 encore.similarity module . . . 6
2.6 encore.utils module . . . 11

3 encore.clustering package 15
3.1 Submodules . . . 15
3.2 encore.clustering.Cluster module . . . 15
3.3 encore.clustering.affinityprop module . . . 16

4 encore.dimensionality_reduction package 17
4.1 Module contents . . . 17

Python Module Index 19

Index 21

CHAPTER

ONE

INTRODUCTION

ENCORE is a Python package designed to quantify the similarity between conformational ensembles of proteins (or in principle other macromolecules), using three different methods originally described in:

```
Kresten Lindorff-Larsen, Jesper Ferkinghoff-Borg (2009)
Similarity Measures for Protein Ensembles.
PLoS ONE 4(1): e4203. doi:10.1371/journal.pone.0004203
```

A description of ENCORE and a number of application can be found in:

```
Matteo Tiberti, Elena Papaleo, Tone Bengtsen, Wouter Boomsma and
Kresten Lindorff-Larsen,
ENCORE: Software for quantitative ensemble comparison
Submitted
```

The package includes facilities for handling ensembles and trajectories, performing clustering or dimensionality reduction of the ensemble space, estimating multivariate probability distributions from the input data, and more. ENCORE can be used to compare experimental and simulation-derived ensembles, as well as estimate the convergence of trajectories from time-dependent simulations. The package was designed as a Python 2.6 (or any higher 2.X version) library. The user may also use some of the library files as scripts that accept command line arguments. Usually, the help text included for each script (obtained running "python encore/script.py -h") is self-explanatory. Examples are also available on how ENCORE may be used to calculate the similarity measures on a number of ensembles.

The similarity measures implemented in ENCORE are based on three different methods, which all rely on the following idea: Given two or more conformational ensembles of the same topology (i.e. structure), we view the particular set of conformations from each ensemble as a sample from an underlying, but unknown, probability distribution. We use this sample to model the probability density function of said distribution. Then we compare the modeled distributions using standard measures of the similarity between two probability densities, such as the Jensen-Shannon divergence.

In the ENCORE package, we have implemented three methods to estimate the density:

- Harmonic ensembles similarity (HES): we assume that each ensemble is derived from a multivariate normal distribution. We, thus, estimate the parameters for the distribution of each ensemble (mean and covariance matrix) and compare them using a symmetrized version of the Kullback-Leibler divergence. For each ensemble, the mean conformation is estimated as the average over the ensemble, and the covariance matrix is calculated by default using a shrinkage estimate method (or by a maximum-likelihood method, optionally).
- Clustering-based similarity (CES): We use the affinity propagation method for clustering to partition the whole space of conformations in to clusters of structures. After the structures are clustered we take the population of each ensemble in each cluster as a probability distribution of conformations. We then compare the obtained probability distribution using the Jensen-Shannon divergence measure between probability distributions.
- Dimensionality reduction-based similarity (DRES): We use a gaussian kernel-based density estimation method to estimate the probability density, and use that as probability function in order to compare different ensembles. Before doing that, however, due to the limited size of the sample, it is necessary to reduce the dimensionality

of the input space. Thus, the method first projects the ensembles into lower dimensions by using the Stochastic Proximity Embedding algorithm.

ENCORE is able to use, as input data, structural ensembles deriving both from molecular simulations (e.g. molecular dynamics or Monte Carlo methods) or experimental structural ensembles (e.g. NMR structures as PDB files). The software is able to handle the most popular trajectory formats (files such as DCD, XTC, TRR, XYZ, TRJ, MDCRD), although periodic boundaries conditions must be removed before use. A topology file is also required.

Together with the software, we also provide three examples that showcase three typical cases of study:

- comparing simulation trajectories with other trajectories
- estimating convergence of trajectories from molecular dynamics simulations
- comparing experimentally-derived ensembles from the PDB

See the examples themselves for more information. If you use ENCORE for your scientific work, please cite:

```
Matteo Tiberti, Elena Papaleo, Tone Bengtsen, Wouter Boomsma
and Kresten Lindorff-Larsen,
ENCORE: Software for quantitative ensemble comparison
Submitted
```

CHAPTER

TWO

ENCORE PACKAGE

2.1 Submodules

2.2 encore.Ensemble module

2.2.1 Ensemble representation — `MDAnalysis.analysis.ensemble.ensemble`

This module contains the Ensemble class allowing for easy reading in and alignment of the ensemble contained in one or more trajectory files. Trajectory files can be specified in several formats, including the popular xtc and dcd, as well as experimental multiple-conformation pdb files, i.e. those coming from NMR structure resoltion experiments.

class `encore.Ensemble.`**`Ensemble`**(*universe=None*, *topology=None*, *trajectory=None*, *atom_selection_string='(name CA)'*, *superimposition_selection_string=None*, *frame_interval=1*)

Ensemble class designed to easily manage more than one trajectory files. Users can provide either a topology/trajectory(es) combination or a MDAnalysis.Universe object. Topology and trajectory files must have the same number of atoms, and order is of course important.

While creating a new Ensemble object it is possible to load from a trajectory a selected subset of atoms, using the MDAnalysis syntax for selections (see http://mdanalysis.googlecode.com/git/package/doc/html/documentation_pages/selections.html for details) and the atom_selection_string argument. By default all the alpha carbons ("CA") are considered. It is also possible to load a lower number of frames for each trajectory, by selecting only one frame every frame_interval (e.g. with frame-interval=2 only every second frame will be loaded).

Frames in an Ensemble object can be superimposed to a reference conformation (see method align). By default the rotation matrix for this superimposition is calculated on all the atoms of the system, as defined by the atom_selection_string. However, if the superimposition_selection_string is provided, that subset will be used to calculate the rotation matrix, which will be applied on the whole atom_selection_string. Notice that the set defined by superimposition_selection_string is completely independent from the atom_selection_string atoms, as it can be a subset or superset of that, although it must refer to the same topology.

Attributes

topology_filename [str] Topology file name.

trajectory_filename [str] Trajectory file name. If more then one are specified, it is a list of comma-separated names (e.g. "traj1.xtc,traj2.xtc")

universe [MDAnalysis.Universe] Universe object containing the original trajectory(es) and all the atoms in the topology.

frame_interval [int] Keep only one frame every frame_interval (see the package or module description)

atom_selection_string [str] Atom selection string in the MDAnalysis format (see http://mdanalysis.googlecode.com/git/package/doc/html/documentation_pages/selections.html)

atom_selection [MDAnalysis.core.AtomGroup] MDAnalysis atom selection, which corresponds to the selection defined by atom_selection_string on universe

coordinates [(x,N,3) numpy.array] Array of coordinate which will be used in the calculations, where x is the number of frames and N is the number of atoms. Notice that these coordinates may be different from those of universe, because of the atom_selection and frame_interval.

superimposition_selection_string [str] Analogous to atom_selection_string, but related to the subset of atoms that will be used for 3D superimposition.

superimposition_selection [MDAnalysis.core.AtomGroup] Analogous to atom_selection, but related to the subset of atoms that will be used for 3D superimposition.

superimposition_coordinates [(x,N,3) numpy.array] Analogous to coordinates, but related to the subset of atoms that will be used for 3D superimposition.

align(*reference=None*, *weighted=True*)
Least-square superimposition of the Ensemble coordinates to a reference structure.

Arguments:

reference [None or MDAnalysis.Universe] Reference structure on which those belonging to the Ensemble will be fitted upon. It must have the same topology as the Ensemble topology. If reference is None, the structure in the first frame of the ensemble will be used as reference.

weighted [bool] Whether to perform weighted superimposition or not

get_coordinates(*subset_selection_string=None*)
Get a set of coordinates from Universe.

Arguments:

subset_selection_string [None or str] Selection string that selects the universe atoms whose coordinates have to be returned. The frame_interval will be automatically applied. If the argument is None, the atoms defined in the atom_selection_string will be considered.

Returns:

coordinates [(x,N,3) numpy array] The requested array of coordinates.

2.3 encore.confdistmatrix module

2.3.1 Distance Matrix calculation — `MDAnalysis.analysis.ensemble.confdistmatrix`

The module contains a base class to easily compute, using parallelization and shared memory, matrices of conformational distance between the structures stored in an Ensemble. A class to compute an RMSD matrix in such a way is also available.

class encore.confdistmatrix.**ConformationalDistanceMatrixGenerator**
Base class for conformational distance matrices generator between array of coordinates. Work for single matrix elements is performed by the private _simple_worker and _fitter_worker methods, which respectively do or don't perform pairwise alignment before calculating the distance metric. The class efficiently and automatically spans work over a prescribed number of cores, while keeping both input coordinates and the output matrix as shared memory. If logging level is low enough, a progress bar of the whole process is printed out. This class acts as a functor.

run (*ensemble*, *ncores=None*, *pairwise_align=False*, *align_subset_coordinates=None*, *mass_weighted=True*, *metadata=True*)
Run the conformational distance matrix calculation.

Arguments:

ensemble [encore.Ensemble.Ensemble object] Ensemble object for which the conformational distance matrix will be computed.

pairwise_align [bool] Whether to perform pairwise alignment between conformations

align_subset_coordinates [numpy.array or None] Use these coordinates for superimposition instead of those from ensemble.superimposition_coordinates

mass_weighted [bool] Whether to perform mass-weighted superimposition and metric calculation

metadata [bool] Whether to build a metadata dataset for the calculated matrix

ncores [int] Number of cores to be used for parallel calculation

Returns:

cond_dist_matrix [encore.utils.TriangularMatrix object] Conformational distance matrix in triangular representation.

class encore.confdistmatrix.**MinusRMSDMatrixGenerator**
Bases: *encore.confdistmatrix.ConformationalDistanceMatrixGenerator*

-RMSD Matrix calculator. See encore.confdistmatrix.RMSDMatrixGenerator for details.

class encore.confdistmatrix.**RMSDMatrixGenerator**
Bases: *encore.confdistmatrix.ConformationalDistanceMatrixGenerator*

RMSD Matrix calculator. Simple workers doesn't perform fitting, while fitter worker does.

2.4 encore.covariance module

2.4.1 Covariance calculation — encore.covariance

The module contains functions to estimate the covariance matrix of an ensemble of structures.

class encore.covariance.**EstimatorML**
Standard maximum likelihood estimator of the covariance matrix. The generated object acts as a functor.

calculate (*coordinates*, *reference_coordinates=None*)
Arguments:

coordinates [numpy.array] Flattened array of coordiantes

reference_coordinates [numpy.array] Optional reference to use instead of mean

Returns:

cov_mat [numpy.array] Estimate of covariance matrix

class encore.covariance.**EstimatorShrinkage** (*shrinkage_parameter=None*)
Shrinkage estimator of the covariance matrix using the method described in

Improved Estimation of the Covariance Matrix of Stock Returns With an Application to Portfolio Selection. Ledoit, O.; Wolf, M., Journal of Empirical Finance, 10, 5, 2003

This implementation is based on the matlab code made available by Olivier Ledoit on his website:

http://www.ledoit.net/ole2_abstract.htm

The generated object acts as a functor.

calculate(*coordinates*, *reference_coordinates=None*)

Arguments:

coordinates [numpy.array] Flattened array of coordiantes

***reference_coordinates*: numpy.array** Optional reference to use instead of mean

Returns:

cov_mat [nump.array] Covariance matrix

encore.covariance.**covariance_matrix**(*ensemble*, *estimator=<encore.covariance.EstimatorShrinkage instance>*, *mass_weighted=True*, *reference=None*, *start=0*, *end=None*)

Calculates (optionally mass weighted) covariance matrix

Arguments:

ensemble [Ensemble object] The structural ensemble

estimator [MLEstimator or ShrinkageEstimator object] Which estimator type to use (maximum likelihood, shrinkage). This object is required to have a __call__ function defined.

mass_weighted [bool] Whether to do a mass-weighted analysis

reference [MDAnalysis.Universe object] Use the distances to a specific reference structure rather than the distance to the mean.

Returns:

cov_mat [numpy.array] Covariance matrix

2.5 encore.similarity module

2.5.1 Ensemble similarity calculations — `encore.similarity`

The module contains implementations of similary measures between protein ensembles described in:

Similarity Measures for Protein Ensembles. Lindorff-Larsen, K.; Ferkinghoff-Borg, J. PLoS ONE 2009, 4, e4203.

encore.similarity.**bootstrap_coordinates**(*coords*, *times*)

Bootstrap conformations in a encore.Ensemble. This means drawing from the encore.Ensemble.coordinates numpy array with replacement "times" times and returning the outcome.

Arguments:

coords [numpy.array] 3-dimensional coordinates array

times [int] number of times the coordinates will be bootstrapped

Returns:

out [list] Bootstrapped coordinates list. len(out) = times.

encore.similarity.**bootstrap_matrix**(*matrix*)

Bootstrap an input square matrix. The resulting matrix will have the same shape as the original one, but the order of its elements will be drawn (with repetition). Separately bootstraps each ensemble.

Arguments:

matrix [encore.utils.TriangularMatrix] similarity/dissimilarity matrix

Returns:

this_m [encore.utils.TriangularMatrix] bootstrapped similarity/dissimilarity matrix

`encore.similarity.`**`clustering_ensemble_similarity`**(*cc*, *ens1*, *ens1_id*, *ens2*, *ens2_id*)

Clustering ensemble similarity: calculate the probability densities from the clusters and calculate discrete Jensen-Shannon divergence.

Arguments:

cc [encore.ClustersCollection] Collection from cluster calculated by a clustering algorithm (e.g. Affinity propagation)

ens1 [encore.Ensemble] First ensemble to be used in comparison

ens2 [encore.Ensemble] Second ensemble to be used in comparison

ens1_id [int] First ensemble id as detailed in the ClustersCollection metadata

ens2_id [int] Second ensemble id as detailed in the ClustersCollection metadata

Returns:

djs [float] Jensen-Shannon divergence between the two ensembles, as calculated by the clustering ensemble similarity method

`encore.similarity.`**`cumulative_clustering_ensemble_similarity`**(*cc*, *ens1*, *ens1_id*, *ens2*, *ens2_id*, *ens1_id_min=1*, *ens2_id_min=1*)

Calculate clustering ensemble similarity between joined ensembles. This means that, after clustering has been performed, some ensembles are merged and the dJS is calculated between the probability distributions of the two clusters groups. In particular, the two ensemble groups are defined by their ensembles id: one of the two joined ensembles will comprise all the ensembles with id [ens1_id_min, ens1_id], and the other ensembles will comprise all the ensembles with id [ens2_id_min, ens2_id].

Arguments:

cc [encore.ClustersCollection] Collection from cluster calculated by a clustering algorithm (e.g. Affinity propagation)

ens1 [encore.Ensemble] First ensemble to be used in comparison

ens2 [encore.Ensemble] Second ensemble to be used in comparison

ens1_id [int] First ensemble id as detailed in the ClustersCollection metadata

ens2_id [int] Second ensemble id as detailed in the ClustersCollection metadata

Returns:

djs [float] Jensen-Shannon divergence between the two ensembles, as calculated by the clustering ensemble similarity method

`encore.similarity.`**`cumulative_gen_kde_pdfs`**(*embedded_space*, *ensemble_assignment*, *nensembles*, *nsamples=None*, *ens_id_min=1*, *ens_id_max=None*)

Generate Kernel Density Estimates (KDE) from embedded spaces and elaborate the coordinates for later use. However, consider more than one ensemble as the space on which the KDE will be generated. In particular, will use ensembles with ID [ens_id_min, ens_id_max].

Arguments:

embedded_space [numpy.array] Array containing the coordinates of the embedded space

ensemble_assignment [numpy.array] array containing one int per ensemble conformation. These allow to distinguish, in the complete embedded space, which conformations belong to each ensemble. For instance if ensemble_assignment is [1,1,1,1,2,2], it means that the first four conformations belong to ensemble 1 and the last two to ensemble 2

nesensembles [int] Number of ensembles

‘nsamples [int] Samples to be drawn from the ensembles. Will be required in a later stage in order to calculate dJS.‘

ens_id_min [int] Minimum ID of the ensemble to be considered; see description

ens_id_max [int] Maximum ID of the ensemble to be considered; see description

Returns:

kdes [scipy.stats.gaussian_kde] KDEs calculated from ensembles

resamples [list of numpy.array] For each KDE, draw samples according to the probability distribution of the kde mixture model

embedded_ensembles [list of numpy.array] List of numpy.array containing, each one, the elements of the embedded space belonging to a certain ensemble

`encore.similarity.`**`dimred_ensemble_similarity`**(*kde1, resamples1, kde2, resamples2, ln_P1_exp_P1=None, ln_P2_exp_P2=None, ln_P1P2_exp_P1=None, ln_P1P2_exp_P2=None*)

Calculate the Jensen-Shannon divergence according the the Dimensionality reduction method. In this case, we have continuous probability densities we have to integrate over the measureable space. Our target is calculating Kullback-Liebler, which is defined as:

$$D_{KL}(P(x)||Q(x)) = \int_{-\infty}^{\infty} P(x_i)ln(P(x_i)/Q(x_i)) = \langle ln(P(x))\rangle_P - \langle ln(Q(x))\rangle_P$$

where the $\langle . \rangle_P$ denotes an expectation calculated under the distribution P. We can, thus, just estimate the expectation values of the components to get an estimate of dKL. Since the Jensen-Shannon distance is actually more complex, we need to estimate four expectation values:

$$\langle log(P(x))\rangle_P$$
$$\langle log(Q(x))\rangle_Q$$
$$\langle log(0.5 * (P(x) + Q(x)))\rangle_P$$
$$\langle log(0.5 * (P(x) + Q(x)))\rangle_Q$$

Arguments:

kde1 [scipy.stats.gaussian_kde] Kernel density estimation for ensemble 1

resamples1 [numpy.array] Samples drawn according do kde1. Will be used as samples to calculate the expected values according to ‘P’ as detailed before.

kde2 [scipy.stats.gaussian_kde] Kernel density estimation for ensemble 2

resamples2 [numpy.array] Samples drawn according do kde2. Will be used as sample to calculate the expected values according to ‘Q’ as detailed before.

ln_P1_exp_P1 [float or None] Use this value for $\langle log(P(x))\rangle_P$; if None, calculate it instead

ln_P2_exp_P2 [float or None] Use this value for $\langle log(Q(x))\rangle_Q$; if None, calculate it instead

ln_P1P2_exp_P1 [float or None] Use this value for $\langle log(0.5 * (P(x) + Q(x)))\rangle_P$; if None, calculate it instead

ln_P1P2_exp_P1 [float or None] Use this value for $\langle log(0.5 * (P(x) + Q(x)))\rangle_Q$; if None, calculate it instead

Returns:

djs [float] Jensen-Shannon divergence calculated according to the dimensionality reduction method

`encore.similarity.`**`discrete_jensen_shannon_divergence`**(*pA*, *pB*)

Jensen-Shannon divergence between discrete probability distributions.

Arguments:

pA [iterable of floats] First discrete probability density function

pB [iterable of floats] Second discrete probability density function

Returns:

djs [float] Discrete Jensen-Shannon divergence

`encore.similarity.`**`discrete_kullback_leibler_divergence`**(*pA*, *pB*)

Kullback-Leibler divergence between discrete probability distribution. Notice that since this measure is not symmetric $d_{KL}(p_A, p_B)! = d_{KL}(p_B, p_A)$

Arguments:

pA [iterable of floats] First discrete probability density function

pB [iterable of floats] Second discrete probability density function

Returns:

dkl [float] Discrete Kullback-Liebler divergence

`encore.similarity.`**`gen_kde_pdfs`**(*embedded_space*, *ensemble_assignment*, *nensembles*, *nsamples=None*, ***kwargs*)

Generate Kernel Density Estimates (KDE) from embedded spaces and elaborate the coordinates for later use.

Arguments:

embedded_space [numpy.array] Array containing the coordinates of the embedded space

ensemble_assignment [numpy.array] Array containing one int per ensemble conformation. These allow to distinguish, in the complete embedded space, which conformations belong to each ensemble. For instance if ensemble_assignment is [1,1,1,1,2,2], it means that the first four conformations belong to ensemble 1 and the last two to ensemble 2

nesensembles [int] Number of ensembles

nsamples : int samples to be drawn from the ensembles. Will be required in a later stage in order to calculate dJS.‘

Returns:

kdes [scipy.stats.gaussian_kde] KDEs calculated from ensembles

resamples [list of numpy.array] For each KDE, draw samples according to the probability distribution of the KDE mixture model

embedded_ensembles [list of numpy.array] List of numpy.array containing, each one, the elements of the embedded space belonging to a certain ensemble

`encore.similarity.`**`harmonic_ensemble_similarity`**(*ensemble1=None*, *ensemble2=None*, *sigma1=None*, *sigma2=None*, *x1=None*, *x2=None*, *mass_weighted=True*, *covariance_estimator=<encore.covariance.EstimatorShrinkage instance>*)

Calculate the harmonic ensemble similarity measure as defined in

Similarity Measures for Protein Ensembles. Lindorff-Larsen, K.; Ferkinghoff-Borg, J. PLoS ONE 2009, 4, e4203.

Arguments:

ensemble1 [encore.Ensemble or None] First ensemble to be compared. If this is None, sigma1 and x1 must be provided.

ensemble2 [encore.Ensemble or None] Second ensemble to be compared. If this is None, sigma2 and x2 must be provided.

sigma1 [numpy.array] Covariance matrix for the first ensemble. If this None, calculate it from ensemble1 using covariance_estimator

sigma2 [numpy.array] Covariance matrix for the second ensemble. If this None, calculate it from ensemble1 using covariance_estimator

***x1*: numpy.array** Mean for the estimated normal multivariate distribution of the first ensemble. If this is None, calculate it from ensemble1

***x2*: numpy.array** Mean for the estimated normal multivariate distribution of the first ensemble.. If this is None, calculate it from ensemble2

mass_weighted [bool] Whether to perform mass-weighted covariance matrix estimation

covariance_estimator [either EstimatorShrinkage or EstimatorML objects] Which covariance estimator to use

Returns:

dhes [float] harmonic similarity measure

`encore.similarity.`**`write_output`**(*matrix*, *base_fname=None*, *header=''*, *suffix=''*, *extension='dat'*)

Write output matrix with a nice format, to stdout and optionally a file.

Arguments:

matrix [encore.utils.TriangularMatrix] Matrix containing the values to be printed

base_fname [str] Basic filename for output. If None, no files will be written, and the matrix will be just printed on screen

header [str] Line to be written just before the matrix

suffix [str] String to be concatenated to basename, in order to get the final file name

extension [str] Extension for the output file

`encore.similarity.`**`write_output_line`**(*value*, *fhandler=None*, *suffix=''*, *label='win.'*, *number=0*, *rawline=None*)

Write a line of data with a fixed format to standard output and optionally file. The line will be appended or written to a file object.

The format is (in the Python str.format specification language): '{:s}{:d} {:.3f}', with the first element being the label, the second being a number that identifies the data point, and the third being the number itself. For instance:

win.3 0.278

Arguments:

value [float] Value to be printed.

fhandler [file object] File object in which the line will be written. if None, nothing will be written to file, and the value will be just printed on screen

label [str] Label to be written before the data

number [int] Number that identifies the data being written in this line.

rawline [str] If rawline is not None, write rawline to fhandler instead of the formatted number line. rawline can be any arbitrary string.

2.6 encore.utils module

class `encore.utils.`**`AllowUnrecognizedOptionParser`**(*usage=None*, *option_list=None*, *option_class=<class optparse.Option>*, *version=None*, *conflict_handler='error'*, *description=None*, *formatter=None*, *add_help_option=True*, *prog=None*, *epilog=None*)

Bases: `optparse.OptionParser`

Parser allowing unknown options. Note that only AmbiguousOptionError is caught, meaning that unexpected arguments to known options still give rise to an error.

class `encore.utils.`**`AnimatedProgressBar`**(**args*, ***kwargs*)

Bases: `encore.utils.ProgressBar`

Extends ProgressBar to allow you to use it straighforward on a script. Accepts an extra keyword argument named *stdout* (by default use sys.stdout). The progress status may be send to any file-object.

`show_progress`()

class `encore.utils.`**`OptionGroup`**(*parser*, *title*, *description=None*)

Bases: `optparse.OptionGroup`

A wrapper for a group of options. Stores the args and kwargs options used to create options within the group, so that duplicates can be made

`add_option`(**args*, ***kwargs*)

`duplicate`(*index*)

Make a copy of the entire group. Any occurrence of "%(index)s" within any of the arguments will be replaced by the provided index

class `encore.utils.`**`OptionGroups`**

Wrapper for the creation of new groups, making it possible to reuse OptionGroup definitions in different parsers (which is normally not possible since they are bound to a specific parser.

`add_group`(*title*)

class `encore.utils.`**`ParallelCalculation`**(*ncores*, *function*, *args=[]*, *kwargs=None*)

Generic parallel calculation class. Can use arbitrary functions, arguments to functions and kwargs to functions.

Attributes:

ncores [int] Number of cores to be used for parallel calculation

function [callable object] Function to be run in parallel.

args [list of tuples] Each tuple contains the arguments that will be passed to function(). This means that a call to function() is performed for each tuple. function is called as function(*args, **kwargs). Runs are distributed on the requested numbers of cores.

kwargs [list of dicts] Each tuple contains the named arguments that will be passed to function, similarly as described for the args attribute.

nruns [int] Number of runs to be performed. Must be equal to len(args) and len(kwargs).

run()
Run parallel calculation.

Returns:

results [tuple of ordered tuples (int, object)] int is the number of the calculation corresponding to a certain argument in the args list, and object is the result of corresponding calculation. For instance, in (3, output), output is the return of function(*args[3], **kwargs[3]).

worker(*q*, *results*)
Generic worker. Will run function with the prescribed args and kwargs.

Arguments:

q [multiprocessing.Manager.Queue object] work queue, from which the worker fetches arguments and messages

results [multiprocessing.Manager.Queue object] results queue, where results are put after each calculation is finished

class encore.utils.**ParserPhase**(*option_groups*, *add_help_option=True*, *allow_unrecognized=False*, *usage=''*)
Wrapper for a parser for a single phase. Takes a list of option groups as arguments

add_option_groups(*option_groups*, *copies=1*)

parse()

class encore.utils.**PassThroughOptionParser**(*usage=None*, *option_list=None*, *option_class=<class optparse.Option>*, *version=None*, *conflict_handler='error'*, *description=None*, *formatter=None*, *add_help_option=True*, *prog=None*, *epilog=None*)
Bases: optparse.OptionParser

An unknown option pass-through implementation of OptionParser.

When unknown arguments are encountered, bundle with largs and try again, until rargs is depleted.

sys.exit(status) will still be called if a known argument is passed incorrectly (e.g. missing arguments or bad argument types, etc.)

class encore.utils.**ProgressBar**(*start=0*, *end=10*, *width=12*, *fill='='*, *blank='.'*, *format='[%(fill)s>%(blank)s] %(progress)s%%'*, *incremental=True*)
Bases: object

Handle and draw a progress barr. From https://github.com/ikame/progressbar

reset()
Resets the current progress to the start point

update(*progress*)
Update the progress value instead of incrementing it

class `encore.utils.`**`Tee`**(**files*)

Simple class that writes to one or more file objects.

Attributes:

files [list of file objects] File objects to be written to.

`write`(*obj*)

Write string obj to all the file objects.

Arguments:

obj [str] String to write to the file objects.

class `encore.utils.`**`TriangularMatrix`**(*size*, *metadata=None*, *loadfile=None*)

Triangular matrix class. This class is designed to provide a memory-efficient representation of a triangular matrix that still behaves as a square symmetric one. The class wraps a numpy.array object, in which data are memorized in row-major order. It also has few additional facilities to conveniently load/write a matrix from/to file. It can be accessed using the [] and () operators, similarly to a normal numpy array.

Attributes:

size [int] Size of the matrix (number of rows or number of columns)

metadata [dict] Metadata for the matrix (date of creation, name of author ...)

`loadz`(*fname*)

Load matrix from the npz compressed numpy format.

Arguments:

fname : str Name of the file to be loaded.

`savez`(*fname*)

Save matrix in the npz compressed numpy format. Save metadata and data as well.

Arguments:

fname : str Name of the file to be saved.

`square_print`(*fname=None*, *header=None*, *label='ens.'*, *justification=10*)

Print the triangular matrix as a symmetrical square matrix. Also supports printing to a file (named fname).

`trm_print`(*justification=10*)

Print the triangular matrix as triangular

`encore.utils.`**`print_square_array`**(*array*)

Pretty print square matrix numpy array

Arguments:

array [numpy.array] numpy array to be printed.

`encore.utils.`**`trm_indeces`**(*a*, *b*)

Generate (i,j) indeces of a triangular matrix, between elements a and b. The matrix size is automatically determined from the number of elements. For instance: trm_indexes((0,0),(2,1)) yields (0,0) (1,0) (1,1) (2,0) (2,1).

Arguments:

a [(int i, int j) tuple] starting matrix element.

b [(int i, int j) tuple] final matrix element.

`encore.utils.`**`trm_indeces_nodiag`**(*n*)

generate (i,j) indeces of a triangular matrix of n rows (or columns), without diagonal (e.g. no elements (0,0),(1,1),...,(n,n))

Arguments:

n [int] Matrix size

encore.utils.**vararg_callback**(*option*, *opt_str*, *value*, *parser*)
A callback for the option parser allowing a variable number of arguments.

CHAPTER

THREE

ENCORE.CLUSTERING PACKAGE

3.1 Submodules

3.2 encore.clustering.Cluster module

3.2.1 Ensemble representation — `MDAnalysis.analysis.ensemble.ensemble`

The module contains the Cluster and ClusterCollection classes which are designed to store results from clustering algorithms.

class `encore.clustering.Cluster.`**`Cluster`**(*elem_list=None*, *centroid=None*, *idn=None*, *metadata=None*)

Generic Cluster class for clusters with centroids.

Attributes:

id [int] Cluster ID number. Useful for the ClustersCollection class

metadata [iterable] dict of lists, containing metadata for the cluster elements. The iterable must return the same number of elements as those that belong to the cluster.

size [int] number of elements.

centroid [element object] cluster centroid.

elements [numpy.array] array containing the cluster elements.

`add_metadata`(*name*, *data*)

class `encore.clustering.Cluster.`**`ClustersCollection`**(*elements=None*, *metadata=None*)

Clusters collection class; this class represents the results of a full clustering run. It stores a group of clusters defined as encore.clustering.Cluster objects.

Attributes:

clusters [list of Cluster objects] clusters object which are part of the Cluster collection

`get_centroids`()

Get the centroids of the clusters

Returns:

centroids [list of cluster element objects] list of cluster centroids

`get_ids`()

Get the ID numbers of the clusters

Returns:

ids [list of int] list of cluster ids

3.3 encore.clustering.affinityprop module

class `encore.clustering.affinityprop.`**`AffinityPropagation`**

Affinity propagation clustering algorithm. This class is a Cython wrapper around the Affinity propagation algorithm, which is implement as a C library (see ap.c). The implemented algorithm is described in the paper:

> Clustering by Passing Messages Between Data Points. Brendan J. Frey and Delbert Dueck, University of Toronto Science 315, 972–976, February 2007

`run`(*self*, *s*, *preference*, *double lam*, *int max_iterations*, *int convergence*, *int noise=1*)

Run the clustering algorithm.

Arguments:

s [encore.utils.TriangularMatrix object] Triangular matrix containing the similarity values for each pair of clustering elements. Notice that the current implementation does not allow for asymmetric values (i.e. similarity(a,b) is assumed to be equal to similarity(b,a))

preference [numpy.array of floats or float] Preference values, which the determine the number of clusters. If a single value is given, all the preference values are set to that. Otherwise, the list is used to set the preference values (one value per element, so the list must be of the same size as the number of elements)

lam [float] Floating point value that defines how much damping is applied to the solution at each iteration. Must be]0,1]

max_iterations [int] Maximum number of iterations

convergence [int] Number of iterations in which the cluster centers must remain the same in order to reach convergence

noise [int] Whether to apply noise to the input s matrix, such there are no equal values. 1 is for yes, 0 is for no.

Returns:

elements [list of int or None] List of cluster-assigned elements, which can be used by encore.utils.ClustersCollection to generate Cluster objects. See these classes for more details.

CHAPTER

FOUR

ENCORE.DIMENSIONALITY_REDUCTION PACKAGE

4.1 Module contents

class `encore.dimensionality_reduction.stochasticproxembed.`**`StochasticProximityEmbedding`**

Stochastic proximity embedding dimensionality reduction algorithm. The algorithm implemented here is described in this paper:

> Dmitrii N. Rassokhin, Dimitris K. Agrafiotis A modified update rule for stochastic proximity embedding Journal of Molecular Graphics and Modelling 22 (2003) 133–140

This class is a Cython wrapper for a C implementation (see spe.c)

`run`(*self*, *s*, *double rco*, *int dim*, *double maxlam*, *double minlam*, *int ncycle*, *int nstep*, *int stressfreq*)

Run stochastic proximity embedding.

Arguments:

s [encore.utils.TriangularMatrix object] Triangular matrix containing the distance values for each pair of elements in the original space.

rco [float] neighborhood distance cut-off

dim [int] number of dimensions for the embedded space

minlam [float] final learning parameter

maxlam [float] starting learning parameter

ncycle [int] number of cycles. Each cycle is composed of nstep steps. At the end of each cycle, the lerning parameter lambda is updated.

nstep [int] number of coordinate update steps for each cycle

Returns:

space [(float, numpy.array)] float is the final stress obtained; the array are the coordinates of the elements in the embedded space

stressfreq [int] calculate and report stress value every stressfreq cycle

class `encore.dimensionality_reduction.stochasticproxembed.`**`kNNStochasticProximityEmbedding`**

k-Nearest Neighbours Stochastic proximity embedding dimensionality reduction algorithm. This is a variation of the SPE algorithm in which neighbourhood is not defined by a distance cut-off; instead, at each step, when a point is randomly chosen to perform coordinate updates, the coordinates of its k nearest neighbours are updated as well. This class is a Cython wrapper for a C implementation (see spe.c)

run (*self*, *s*, *int kn*, *int dim*, *double maxlam*, *double minlam*, *int ncycle*, *int nstep*, *int stressfreq*)

Run kNN-SPE.

Arguments:

s [encore.utils.TriangularMatrix object] Triangular matrix containing the distance values for each pair of elements in the original space.

kn [int] number of k points to be used as neighbours, in the original space

dim [int] number of dimensions for the embedded space

minlam [float] final learning parameter

maxlam [float] starting learning parameter

ncycle [int] number of cycles. Each cycle is composed of nstep steps. At the end of each cycle, the lerning parameter lambda is updated.

nstep [int] number of coordinate update steps for each cycle

Returns:

space [(float, numpy.array)] float is the final stress obtained; the array are the coordinates of the elements in the embedded space

stressfreq [int] calculate and report stress value every stressfreq cycle

PYTHON MODULE INDEX

e

encore.clustering.affinityprop, 16
encore.clustering.Cluster, 15
encore.confdistmatrix, 4
encore.covariance, 5
encore.dimensionality_reduction, 17
encore.dimensionality_reduction.stochasticproxembed, 17
encore.Ensemble, 3
encore.similarity, 6
encore.utils, 11

A

add_group() (encore.utils.OptionGroups method), 11
add_metadata() (encore.clustering.Cluster.Cluster method), 15
add_option() (encore.utils.OptionGroup method), 11
add_option_groups() (encore.utils.ParserPhase method), 12
AffinityPropagation (class in encore.clustering.affinityprop), 16
align() (encore.Ensemble.Ensemble method), 4
AllowUnrecognizedOptionParser (class in encore.utils), 11
AnimatedProgressBar (class in encore.utils), 11

B

bootstrap_coordinates() (in module encore.similarity), 6
bootstrap_matrix() (in module encore.similarity), 6

C

calculate() (encore.covariance.EstimatorML method), 5
calculate() (encore.covariance.EstimatorShrinkage method), 6
Cluster (class in encore.clustering.Cluster), 15
clustering_ensemble_similarity() (in module encore.similarity), 7
ClustersCollection (class in encore.clustering.Cluster), 15
ConformationalDistanceMatrixGenerator (class in encore.confdistmatrix), 4
covariance_matrix() (in module encore.covariance), 6
cumulative_clustering_ensemble_similarity() (in module encore.similarity), 7
cumulative_gen_kde_pdfs() (in module encore.similarity), 7

D

dimred_ensemble_similarity() (in module encore.similarity), 8
discrete_jensen_shannon_divergence() (in module encore.similarity), 9
discrete_kullback_leibler_divergence() (in module encore.similarity), 9
duplicate() (encore.utils.OptionGroup method), 11

E

encore.clustering.affinityprop (module), 16
encore.clustering.Cluster (module), 15
encore.confdistmatrix (module), 4
encore.covariance (module), 5
encore.dimensionality_reduction (module), 17
encore.dimensionality_reduction.stochasticproxembed (module), 17
encore.Ensemble (module), 3
encore.similarity (module), 6
encore.utils (module), 11
Ensemble (class in encore.Ensemble), 3
EstimatorML (class in encore.covariance), 5
EstimatorShrinkage (class in encore.covariance), 5

G

gen_kde_pdfs() (in module encore.similarity), 9
get_centroids() (encore.clustering.Cluster.ClustersCollection method), 15
get_coordinates() (encore.Ensemble.Ensemble method), 4
get_ids() (encore.clustering.Cluster.ClustersCollection method), 15

H

harmonic_ensemble_similarity() (in module encore.similarity), 9

K

kNNStochasticProximityEmbedding (class in encore.dimensionality_reduction.stochasticproxembed), 17

L

loadz() (encore.utils.TriangularMatrix method), 13

M

MinusRMSDMatrixGenerator (class in encore.confdistmatrix), 5

O

OptionGroup (class in encore.utils), 11

OptionGroups (class in encore.utils), 11

P

ParallelCalculation (class in encore.utils), 11
parse() (encore.utils.ParserPhase method), 12
ParserPhase (class in encore.utils), 12
PassThroughOptionParser (class in encore.utils), 12
print_square_array() (in module encore.utils), 13
ProgressBar (class in encore.utils), 12

R

reset() (encore.utils.ProgressBar method), 12
RMSDMatrixGenerator (class in encore.confdistmatrix), 5
run() (encore.clustering.affinityprop.AffinityPropagation method), 16
run() (encore.confdistmatrix.ConformationalDistanceMatrixGenerator method), 4
run() (encore.dimensionality_reduction.stochasticproxembed.kNNStochasticProximityEmbedding method), 17
run() (encore.dimensionality_reduction.stochasticproxembed.StochasticProximityEmbedding method), 17
run() (encore.utils.ParallelCalculation method), 12

S

savez() (encore.utils.TriangularMatrix method), 13
show_progress() (encore.utils.AnimatedProgressBar method), 11
square_print() (encore.utils.TriangularMatrix method), 13
StochasticProximityEmbedding (class in encore.dimensionality_reduction.stochasticproxembed), 17

T

Tee (class in encore.utils), 12
TriangularMatrix (class in encore.utils), 13
trm_indeces() (in module encore.utils), 13
trm_indeces_nodiag() (in module encore.utils), 13
trm_print() (encore.utils.TriangularMatrix method), 13

U

update() (encore.utils.ProgressBar method), 12

V

vararg_callback() (in module encore.utils), 14

W

worker() (encore.utils.ParallelCalculation method), 12
write() (encore.utils.Tee method), 13
write_output() (in module encore.similarity), 10
write_output_line() (in module encore.similarity), 10
